# The multifaceted roles of ER and Golgi in metabolic cardiomyopathy

**DOI:** 10.3389/fcvm.2022.999044

**Published:** 2022-09-02

**Authors:** Rida Raja, Oveena Fonseka, Haresh Ganenthiran, Andrea-Ruiz-Velasco, Wei Liu

**Affiliations:** Faculty of Biology, Medicine, and Health, The University of Manchester, Manchester, United Kingdom

**Keywords:** metabolic syndrome, metabolic cardiomyopathy, cardiac lipid metabolism, endoplasmic reticulum, Golgi apparatus

## Abstract

Metabolic cardiomyopathy is a significant global financial and health challenge; however, pathophysiological mechanisms governing this entity remain poorly understood. Among the main features of metabolic cardiomyopathy, the changes to cellular lipid metabolism have been studied and targeted for the discovery of novel treatment strategies obtaining contrasting results. The endoplasmic reticulum (ER) and Golgi apparatus (GA) carry out protein modification, sorting, and secretion activities that are more commonly studied from the perspective of protein quality control; however, they also drive the maintenance of lipid homeostasis. In response to metabolic stress, ER and GA regulate the expression of genes involved in cardiac lipid biogenesis and participate in lipid droplet formation and degradation. Due to the varied roles these organelles play, this review will focus on recapitulating the alterations and crosstalk between ER, GA, and lipid metabolism in cardiac metabolic syndrome.

## Introduction

In the past five decades, the global prevalence of obesity, diabetes, and hypertension has reached epidemic dimensions (1, 2). These disorders, collectively termed metabolic syndrome, confer substantial morbidity and mortality of cardiovascular disease (3, 4). At the same time, they are high-risk factors for the development of cardiomyopathy (5). The metabolic syndrome encompasses a diverse set of conditions that exert chronic stress on cellular functions, such as systemic dyslipidemia, hyperglycemia, and insulin resistance. The pathophysiological changes induced in the heart by these stressors are termed metabolic cardiomyopathy. Clinically, patients with metabolic cardiomyopathy initially present diastolic dysfunction, followed by late-onset systolic dysfunction and, ultimately, heart failure (HF) (6). Along with adverse structural remodeling and increased presence of reactive oxygen species, cell death, and inflammation (7, 8), metabolic cardiomyopathy features cardiac energetic impairment (9, 10). Lipid mishandling and subsequent lipotoxicity are prominent effects of this alteration (11, 12). Efficacious therapeutic options targeting cardiac lipid derangements are lacking, therefore advanced understanding of the pathogenesis is necessary. The endoplasmic reticulum (ER) and the Golgi apparatus (GA) are essential for lipid homeostasis, hosting and processing proteins involved in triacylglycerol (TG) synthesis and lipolysis. Previous reviews have focused on the central role the mitochondria play in lipid oxidation in metabolic cardiomyopathy (13–16); however, the role of the ER and GA in lipid metabolism in the heart under metabolic stress remains poorly defined.

## Cardiac lipid metabolism

Fatty acids (FAs) account for approximately 70% of the energy sources required for ATP synthesis in cardiomyocytes. In circulation, FAs are commonly found as triacylglycerols (TGs) associated with lipoproteins or chylomicrons or as free FAs bound to albumin. TGs are hydrolyzed by lipoprotein lipase (LpL) Cardiomyocytes take up extracellular FAs by passive diffusion across the plasma membrane or by surface receptors such as protein cluster of differentiation 36 (CD36), fatty acid transport protein 1, and fatty acid binding protein (17). Once in the cytoplasm, FAs are shuttled into the mitochondria for β-oxidation resulting in high energy molecules required for cardiac function (18). Excessive FAs are converted back to TGs by diacylglycerol acyltransferase (DGAT) and stored in lipid droplets (LD). When required, FAs are released from LDs by adipocyte triglyceride lipase (ATGL) and hormone-sensitive lipase (HSL) (19). Lipid homeostasis also requires the participation of other organelles. For example, peroxisomes manage very long-chain fatty acid breakdown and β-oxidation, lysosomes carry out lipid catabolism, and ER and GA handle LD formation (20). It is widely accepted that in metabolic syndrome the myocardium experiences an increase in lipid consumption (9, 21), meaning that different organelles activate stress responses to manage different stages of lipid homeostasis.

## Lipotoxicity in cardiomyopathy

Cardiac lipotoxicity develops due to a build-up of FAs and lipid intermediates that disrupt β-oxidation homeostasis. Among such intermediates are ceramides and di-acyl glycerol (DAG). In a mouse model of lipid-induced dilated cardiomyopathy by LpL cardiac-overexpression, ceramides were found to regulate substrate utilization, where inhibition of ceramide biosynthesis normalized FAs and glucose oxidation and improved survival. In addition, ceramides induce various pathological processes that promote lipotoxicity, for instance, insulin resistance, inflammation, and apoptosis (22).

Similarly, DAG induces apoptosis via mitochondrial apoptotic pathways. It also impairs insulin signaling, an effect that can be accelerated due to the reactive oxygen species production (ROS) by free FAs (19). These pathological processes associated with toxic lipid intermediates contribute to the development of metabolic cardiomyopathy; therefore, it is important to study the mechanisms available in the cell to counteract such conditions.

## ER stress response and UPR

The ER is involved in protein quality control (PQC), calcium homeostasis, and lipid metabolism. Chronic metabolic stress can perturb ER homeostasis leading to ER stress and proteotoxicity that consist of protein misfolding and protein accumulation. Protein quality is vital to sustain cardiac function and in metabolic cardiomyopathy it has been identified as an inducer of cell death (23). ER stress triggers an adaptive signal transduction pathway known as the unfolded protein response (UPR) which neutralizes ER stress and sustains cellular function. Three transmembrane proteins maintain ER homeostasis; inositol- requiring enzyme 1α (IRE1α), protein kinase RNA-like ER kinase (PERK), and activating transcription factor 6 (ATF6) initiate the UPR (24). Under basal conditions, these sensor proteins are associated with chaperone GRP78/Bip, rendering them inactive (25). When proteostasis is disrupted, GRP78/Bip detaches and these become active (26). IRE1α processes the transcription factor X-box binding protein 1 (XBP1), forming the transcriptionally active spliced-XBP1 (XBP1s) (25, 27). XBP1s binds to a set of UPR-target genes that upregulate organelle biosynthesis, PQC, and ER-associated degradation (ERAD) (28). PERK phosphorylates the eukaryotic translation initiating factor 2α (elf2α), attenuating protein synthesis (27, 29). In turn, elf2α also induces the translation of ATF4, which regulates the expression of genes involved in autophagy, apoptosis, antioxidant response, and the transcription factor DNA damage-inducible 34 (GADD34). GADD34 can restore protein synthesis by binding to protein phosphatase 1C (PP1C), which dephosphorylates elf2α (27). PERK upregulates the proapoptotic transcription factor CHOP/GADD153. Finally, ATF6's transcriptional activity induces the expression of ERAD and XBP1 genes (24). These transcriptional events act in a well-choreographed manner to maintain ER equilibrium.

## ER regulation of lipid metabolism

**Figure 1:**
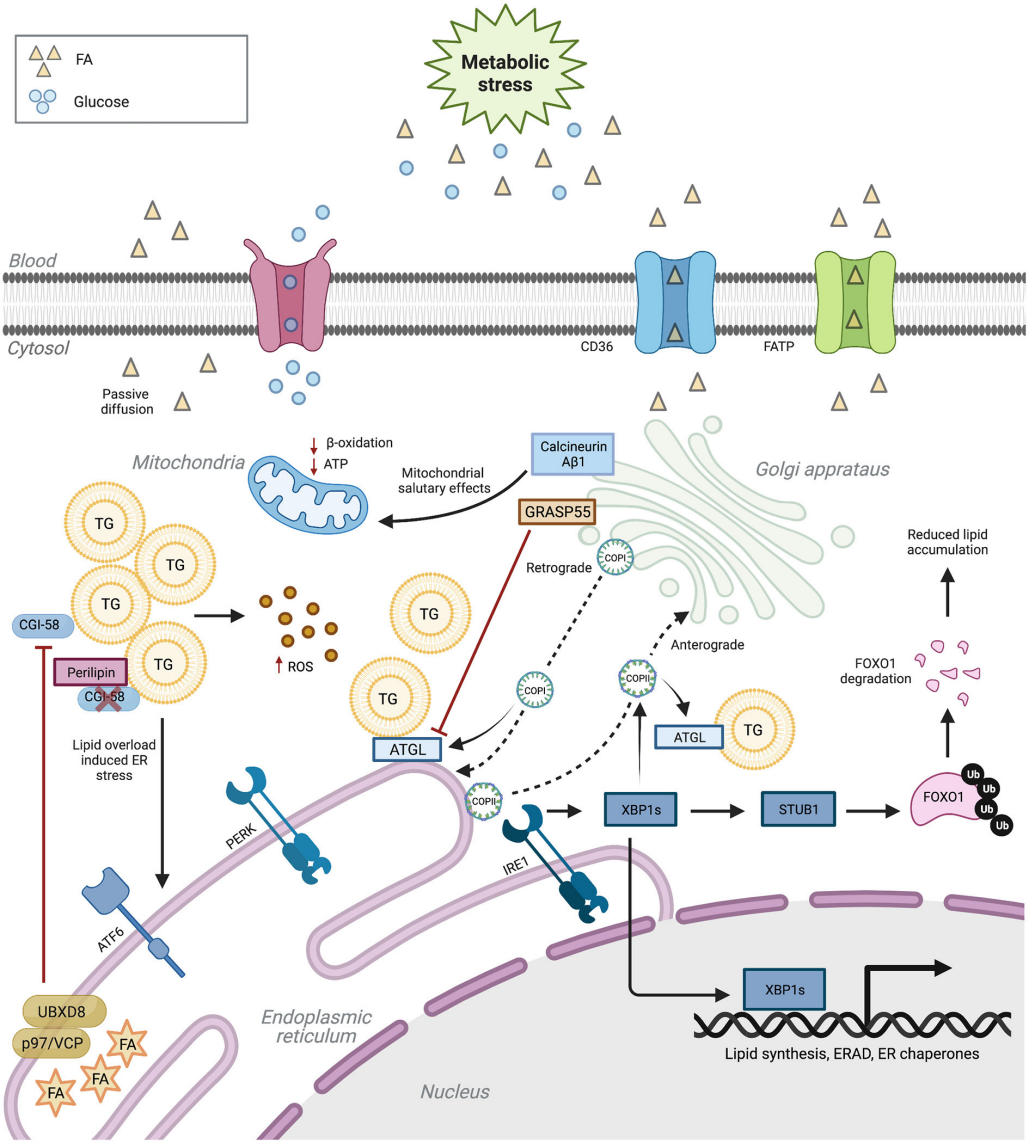
ER and Golgi regulation of lipid metabolism. ER and Golgi functions are paramount for lipid metabolism. Aberrant ER and Golgi function induce cellular lipid disequilibrium. In turn, lipotoxicity negatively regulates the function of these two organelles. Upon metabolic stress, ER dysfunction inhibits TG degradation, eventually leading to oxidative stress. ER stress also reduces genes participating in ER function and lipid metabolism. Impaired Golgi proteins or COPI/II pathways damage lipogenesis and lipolysis balance. CD36 (cluster of differentiation 36), FATP (fatty acid transport protein), TG (triacylglycerol), FA (fatty acid), ROS (reactive oxygen species), ATGL (adipose triglyceride lipase), XBP1s (spliced X-box binding protein 1), FoxO1 (Forkhead box protein O1), COPI (coat protein complex I) and COPII (coat protein complex II). Created with Biorender.com.

In addition to its protein-centric role, the UPR is an essential nutrient sensing ER apparatus critical for maintaining lipid homeostasis (30, 31). Palmitate activates the PERK-eIF2α-CHOP pathway and decreases Bip expression in HepG2 liver cells. Accordingly, Bip overexpression reduced CHOP levels and attenuated palmitate-induced ER stress and apoptosis (32). Moreover, GADD34-mediated dephosphorylation of eIF2α reduces hepatosteatosis in *Alb::GC* transgenic mice upon high-fat diet (HFD) feeding (33). Following this, depletion of ATF4, the downstream effector of the PERK-eIF2α pathway, protects mice against hepatic steatosis and hypertriglyceridemia in response to high-fructose diet feeding (34), suggesting that the PERK pathway regulates lipogenesis in hepatocytes. Similarly, PERK depletion inhibits lipogenesis during the differentiation of mouse embryonic fibroblasts to adipocytes (35) (Figure 1).

The IRE1α-XBP1 branch of the UPR is also a critical regulator of metabolic-stress-induced lipid disequilibrium (36). Adipocyte Xbp1s overexpression prevents obesity in HFD-fed mice and leptin-deficient *ob/ob* mice via increased uridine biosynthesis (37). Added to this, ectopic adipocyte Xbp1s expression promotes systemic glucose homeostasis in both lean and *ob/ob* mice by increasing adiponectin serum levels (38). XBP1 deletion reduces hepatic steatosis and improves insulin sensitivity in mice fed a high-fructose diet despite increased ER stress (39). In accordance, Lee et al. (40) demonstrated that XBP1 regulates lipogenesis. Finally, ATF6 modulates lipid metabolism in an XBP1-independent manner (41). ATF6α-knockout mice administered the pharmacological ER-stress inducer tunicamycin exhibit liver dysfunction and steatosis, ensued from aberrant ß-oxidation and suppression of very-low-density lipoproteins (VLDLs) formation. However, these abnormalities are obliterated by ATF6 overexpression (42). A similar account is observed in ATF6α deficient hepatocytes following a high-fat and high-sucrose diet feeding (43). During adipogenesis, ATF6α deficiency reduces the expression of key audiogenic genes needed for adipocyte differentiation (44). These studies highlight the importance of the ER stress response regulating lipid homeostasis in metabolically active peripheral tissues, such as adipose and liver. The crosstalk between such tissues and the heart is a major subject of study and their influence in the development of metabolic cardiomyopathy has been reported (45, 46). However, the amount of evidence also suggests that similar mechanisms could be present in the heart and be worth exploring for a better understanding of the disease.

## ERAD factors' involvement in lipid metabolism

ERAD is an integral part of the ER stress response. It recognizes and labels abnormal proteins in the ER directing them to the cytosol for proteasomal degradation. Cell-type-specific ERAD mouse model studies are limited. Adipocyte-specific Sel1L deficient mice are resistant to HFD-induced obesity, developing postprandial hypertriglyceridemia and displaying hepatic steatosis (47). Here, in the absence of Sel1L, LPL is retained in the ER in the form of ERAD-resistant aggregates. Similar observations were made on other LPL-expressing cells, including cardiomyocytes and macrophages (47). Hepatocyte-specific ER degradation enhancing alpha-mannosidase-like protein 3 (EDEM3) knockdown mice had increased LRP1 expression leading to enhanced VLDL uptake, thereby reducing plasma TG levels (48). Concurrently, data obtained from Gan et al. (49) showed that ischemic heart-derived small extracellular vesicles (sEVs) carrying miR-23-27-24 suppress adiponectin biosynthesis by downregulating EDEM3. This pathological communication causes adipocyte ER stress and endocrine dysfunction, which contributed to post-MI metabolic disorders.

Furthermore, Choi et al. (50) reported that depletion of the ER membrane-anchored E3 ligase gp78 stabilized DGAT2 in hepatic cells, which increased LD levels. UBXD8 is a protein that interacts with p97 during ERAD to facilitate proteasomal degradation of ubiquitinated proteins; however, UBXD8 has also emerged as a critical determinant of fatty acid (FA) metabolism and TG storage (51–54). UBXD8 acts as an unsaturated FA sensor. In FA-depleted cultured cells, UBXD8 inhibits the conversion of DAGs to TGs; this effect is reversed upon exposure to unsaturated FAs (53). Mechanistically, UBXD8 negatively regulates ATGL by promoting the dissociation of its activator CGI-58 (51). Therefore, FAs increase TG content by inactivating UBXD8. In line with this, UBXD8 depletion in murine hepatocytes leads to periportal steatosis upon HFD feeding (55). Whilst there is a vast body of literature evidencing ERAD involvement in lipid metabolism, the role of ERAD proteins in metabolic cardiomyopathy remains unexplored.

## ER stress and cardiac lipid metabolism

ER stress has been identified as a crucial pathophysiological driver of cardiovascular disease in preclinical and clinical studies (23). ER stress is implicated in cardiac lipotoxicity (56–58); however, the mechanisms by which ER disruptions contribute to lipotoxic alterations in cardiomyocytes are unclear. Recently, Schiattarella et al. (59) reported that cardiomyocyte-specific overexpression of Xbp1s ameliorates cardiac dysfunction and reduces myocardial steatosis in a preclinical model of HF with preserved ejection fraction. Further investigations revealed that Xbp1s promotes the ubiquitination and degradation of Forkhead box protein O1 (FoxO1) via E3 ubiquitin ligase STUB1. FoxO1 is a vital transcriptional regulator of genes involved in cellular metabolism and its transcriptional activity is increased in obese and diabetic animal models (60, 61). In cardiac muscle cells, FoxO1 orchestrates lipid accumulation; the sequential events underpining this accumulation remain unknown. Conversley, FoxO1 overexpression inhibits lipid accumulation in hepatocytes by mediating ATGL-dependent lipolysis (62). CGI-58 is an LD-associated protein that plays a vital role in TG hydrolysis by activating ATGL (63). it is decreased in failing human hearts (64). CGI-58-deficient mice display adverse cardiac remodeling accompanied by accentuated cardiac TG levels, ROS production, and inflammation when after HFD feeding (65). The chemical chaperone 4-PBA attenuated HFD-induced cardiac remodeling and dysfunction; alleviated mitochondrial dissonance, oxidative stress, and lipid accumulation in cardiac-specific CGI58- knockout mice (65). Moreover, inhibiting calpains, calcium-activated proteases, prevents lipotoxicity-associated myocardial injury in HFD-challenged mice by attenuating ER stress, thereby improving cardiac function (66). These studies suggest ER stress regulates cardiac lipid homeostasis and acts as a potential therapeutic target for metabolic cardiomyopathy.

While ER stress alters lipid metabolism, FAs can also induce ER stress in a bidirectional loop, creating a vicious cycle (67). Saturated fatty acids instigate lipotoxic injury in cardiomyocytes via ER stress-mediated apoptosis pathways (68–70). Cardiomyocyte-specific PPARβ/δ deletion perturbed myocardial fatty acid oxidation, induced ER stress, and lead to cardiac steatosis, hypertrophy, and congestive HF in a preclinical mouse model (71, 72). The PPARβ/δ agonist, GW501516, attenuated palmitate-induced ER-stress in AC16 cells, underscoring the therapeutic potential of PPARβ/δ for ER-stress mediated cardiac lipotoxicity (72). Moreover, CTRP9, a highly conserved paralog of adiponectin, has also been reported to regulate lipid metabolism. CTRP9-knockout mice displayed augmented ER-stressed induced apoptosis; nevertheless, recombinant CTRP9 treatment exerted anti-ER-stress-related apoptotic effects and anti-oxidative stress effects to abate lipotoxicity in neonatal rat cardiomyocytes (73).

## The physiological function of the GA

The GA comprises a series of stacked membranes that process ER proteins and lipids. Its main functions include protein glycosylation, sorting and secretion, and phospholipid and sphingomyelin synthesis. The GA network can be divided into the cis and trans components. While the cis network is responsible for receiving cargo from the ER, the trans-Golgi network (TGN), on the other end, regulates cargo exit from the Golgi. The cargo is carried in transport vesicles formed by protein complexes that regulate their content and direction. From the ER exit sites, coat protein complex II (COPII) vesicles travel through the ER-Golgi intermediate compartment (ERGIC) to the cis-component via anterograde transport. In retrograde transport, COPI-coated vesicles recycle ER-resident proteins. Finally, cargo directed to other organelles or the plasma membrane exit the TGN in clathrin-covered vesicles (74). Like the ER, the GA can experience stress when unable to keep up with the flux of proteins and lipids, leading to the activation of the Golgi stress response. Even though this response has not been as well-characterized as the UPR in the heart, pathways such as transcription factor binding to IGHM enhancer 3 (TFE3), CAMP responsive element binding protein 3 (CREB3), and heat shock protein 47 (HSP47) have been found to regulate GA structure and functions (75).

## GA regulation of lipid metabolism

The GA is mainly known for its role in lysosomal, transmembrane, and secretory protein glycosylation and trafficking activities. In addition to its protein processing activities, its contributions to lysosomal function and membrane dynamics are vital for lipid recycling, lysosomal lipid signaling, and membrane composition, all of which are key to lipid metabolism in the setting of metabolic disease (76–78). Presently, manifestations of direct GA involvement in lipid signaling and metabolism not associated to membrane dynamics that have not been clearly defined will be discussed. Recent observations from Fan et al. (79) demonstrated that hyperglycemia affects myocardial GA protein expression and causes fragmentation in a preclinical model of type 1 diabetes. Proofs of how this could impair lipid metabolism have been found in different animal models or cell lines. For instance, the membrane lipid transporter CD36 is processed in the GA before its transport to the plasmatic membrane. A mutation that blunted its passing through the GA resulted in ER accumulation and low lipid uptake (80).

Knocking down COPI subunits in flies (81) and Hep62 (82) cells showed they are required for lipolysis activation. They regulate LD surface proteins that inhibit lipolysis, such as Perilipins 2 and 3, and promote the formation of ER-LD bridges through which ATGL is transferred to the LDs. The deletion of oxysterol binding protein-like 2 (OSBPL2), an intracellular lipid receptor, disrupted LD localization of the subunit COPBI, leading to lipid accumulation in larger LDs and hypercholesterolemia in zebrafish (82). Furthermore, genetic or chemical interference with the ADP-ribosylation factor (ARF1)-COPI pathway at different stages gave similar results. Golgi-specific brefeldin A-resistance guanine nucleotide exchange factor 1 (GBF1) interacts directly with ATGL (83), and its deletion hindered ATGL transport to nascent LDs, inducing the lipase's proteasomal degradation (84). In addition, impeding the function of the COPII complex also disrupted lipolysis, but the effect was smaller than that of COPI. In different cells, knockdown of ARF1, a COPI complex initiator, showed that this machinery has the ability to form nano-buds from the LD membranes and target TG synthesis enzyme to the LD membrane (85), suggesting that it affects LD composition as well as degradation.

Similar to the observations above, deletion of GRASP55, another Golgi-resident protein, impaired the trafficking of ATGL and MGL to LDs, impairing lipolysis. In mice, this systemic deletion conferred resistance to HFD-induced obesity due to a decreased formation and secretion of chylomicrons and VLDLs from enterocytes resulting in low lipid uptake (86). Hepatic deletion of the initiator of COPII complex GTP-binding protein SAR1b (SAR1) in mice showed increased accumulation of TGs and cholesterol in the liver with reduced lipid plasma levels protecting them from atherosclerosis (87). In this study, Wang et al. (87) identified surfeit locus protein 4 (SURF4) as a receptor for specific VLDL secretion downstream of the COPII pathway and as a protein with a COPI sorting signal for recycling. Comparable effects were observed in liver-specific IRE1 knockout mice where COPII transport was reduced. In this case, XBP1 overexpression restored COPII gene expression and trafficking, resolving the fatty liver and hypolipidemia (88). The significance of the GA transport role in lipoprotein secretion was confirmed by a report showing eight different mutations of *SARA2* (coding SAR1 protein) in patients with severe fat absorption disorders (89). These results indicate that the ER-GA transport network can be targeted for the systemic regulation of metabolic diseases.

Finally, in addition to its role in protein and LD processing, the GA is involved in metabolic and cell death signaling. In the heart, activating an alternative isoform of calcineurin presenting a new Golgi-localization signal was found to regulate metabolism through AKT phosphorylation, reducing mitochondrial dysfunction and preventing myocardial remodeling following transaortic constriction (90). AKT signaling is impaired in the hearts of obese mice (91), and this pathway inhibits cardiac lipolysis (92). Furthermore, GA stress pathways have been found to promote apoptosis, particularly in neurological diseases, by modulating caspase cleavage and calcium influx (93). In Hela cells, the ferroptosis pathway was also activated by inducers of GA stress (94). Ferroptosis is an iron-dependent cell death process driven by lipid peroxidation, making these observations relevant for the study of GA and metabolic stress. There is clear evidence that the GA regulates lipid metabolism and that it responds to stress in relevant metabolically active tissues. However, the specific links involved in metabolic cardiomyopathy, particularly taking place in the myocardium, need further study to identify better potential therapeutic targets.

## Conclusion

The ER and GA regulation of lipid metabolism are complex and largely unexplored. Both organelles have a clear role in sustaining protein synthesis and processing during metabolic stress conditions, which is essential for preventing HF. However, their part goes beyond protein quality control; they are deeply involved in different stages of lipid metabolism, from synthesis to storage and catabolism, by processing lipids and governing lipid droplet dynamics. Moreover, their sensing abilities trigger signaling pathways that coordinate their own functional capacity and that of other organelles. In metabolic cardiomyopathy, these tasks become even more critical due to the altered lipid profile state and the high lipotoxicity risks. Therefore, it follows that further studying and targeting ER and GA homeostasis for the treatment of metabolic cardiomyopathy could provide opportunities for the prevention of HF.

## Author contributions

RR, OF, and AR-V collected references, drafted, and proofread the manuscript. RR and HG generated the figure. AR-V and WL designed the manuscript. All authors contributed to the article and approved the submitted version.

## Funding

This work as supported by grants FS/15/16/31477, FS/18/73/33973, PG/19/66/34600, and FS/19/70/34650 to WL from the British Heart Foundation.

## Conflict of interest

The authors declare that the research was conducted in the absence of any commercial or financial relationships that could be construed as a potential conflict of interest.

## Publisher's note

All claims expressed in this article are solely those of the authors and do not necessarily represent those of their affiliated organizations, or those of the publisher, the editors and the reviewers. Any product that may be evaluated in this article, or claim that may be made by its manufacturer, is not guaranteed or endorsed by the publisher.
